# Retrosigmoid approach for giant cystic vestibular schwannoma: subperineural dissection technique for facial nerve preservation

**DOI:** 10.3171/2021.7.FOCVID21128

**Published:** 2021-10-01

**Authors:** Ali Tayebi Meybodi, Robert W. Jyung, James K. Liu

**Affiliations:** Departments of 1 Neurological Surgery and; 2 Otolaryngology–Head and Neck Surgery,; 3 Center for Skull Base and Pituitary Surgery, Rutgers University New Jersey Medical School, Newark; Saint Barnabas Medical Center, RWJ Barnabas Health, Livingston, New Jersey

**Keywords:** vestibular schwannoma, acoustic neuroma, retrosigmoid approach, facial nerve preservation, subperineural dissection technique, operative video, technical nuances

## Abstract

In this illustrative video, the authors demonstrate retrosigmoid resection of a giant cystic vestibular schwannoma using the subperineural dissection technique to preserve facial nerve function. This thin layer of perineurium arising from the vestibular nerves is used as a protective buffer to shield the facial and cochlear nerves from direct microdissection trauma. A near-total resection was achieved, and the patient had an immediate postoperative House-Brackmann grade I facial nerve function. The operative nuances and pearls of technique for safe cranial nerve and brainstem dissection, as well as the intraoperative decision and technique to leave the least amount of residual adherent tumor, are demonstrated.

The video can be found here: https://stream.cadmore.media/r10.3171/2021.7.FOCVID21128

## Transcript

This is Dr. James Liu, and I will be demonstrating the subperineural dissection technique for microsurgical resection of a giant cystic vestibular schwannoma via the retrosigmoid approach.

### 0:32 Patient History and Neurological Examination

The patient is a 50-year-old male, who presented with worsening gait ataxia, severe imbalance, vertigo, and dizziness, associated with worsening facial numbness and left-sided hearing loss. Neurological exam was significant for left facial numbness in V1 to V3, left sensorineural hearing loss, and inability to perform tandem gait.

### 0:51 Preoperative Imaging and Audiogram

MRI scan demonstrated a large, partially cystic enhancing mass in the left cerebrellopontine angle with some intracanalicular extension, measuring greater than 4 cm. This was most consistent with a giant cystic medial vestibular schwannoma.^1–3^ There was significant compression of the brainstem with effacement of the fourth ventricle resulting in obstructive hydrocephalus. The preoperative audiogram showed a left pure tone average of 15 dB and word discrimination score of 88%, consistent with class A hearing.

### 1:26 Treatment Strategy and Choice of Surgical Approach

Given the large size of the tumor with symptomatic brainstem compression, observation and radiosurgery are not recommended. Microsurgical resection is the best option in this case to decompress the brainstem and possibly relieve hydrocephalus without permanent shunting. The potential surgical routes considered here are the retrosigmoid and translabyrinthine approaches. The middle fossa approach is not indicated here since this is reserved for intracanlicular tumors with serviceable hearing. We eventually chose the retrosigmoid approach because of the preoperative hearing status and the vast majority of tumor within the CP angle. Our primary goals of surgery were to perform maximal safe resection with facial nerve preservation and brainstem decompression. Hearing preservation is often challenging in giant cystic tumors. Nevertheless, we make all attempts to preserve the cochlear nerve anatomically, so that cochlear implant can be an option if hearing is not preserved.

### 2:27 Lateral Position and Skin Incision

The patient is placed in the lateral park-bench position with the head in three-pin fixation. The head is positioned so that the nose is pointing slightly toward the floor. Image guidance is used and a retroauricular C-shaped incision is marked about three finger breadths behind the pinna. Facial nerve EMG, facial motor evoked potential, auditory brainstem responses, and long-tract motor evoked potentials are monitored. An external ventricular drain was placed intraoperatively.

### 2:54 Soft-Tissue Exposure and Retrosigmoid Craniectomy

A galeocutaneous flap is elevated and the splenius capitis muscle is mobilized inferiorly. A retrosigmoid craniectomy is performed to decompress the transverse and sigmoid sinuses. The mastoid air cells are then waxed off. A reverse C-shaped dural incision is made, and the dural leaflets are tacked up with sutures.

### 3:15 Intradural Exposure and Tumor Removal

The CSF is released from the lower cervicomedullary cisterns to allow brain relaxation. The spinal accessory nerve is now visualized. The arachnoid membranes are carefully peeled away from the tumor capsule to facilitate medial mobilization of the cerebellum. The dorsal aspect of the tumor is stimulated with a Prass probe to ensure that there is no posterior course of the facial nerve.

After this has been confirmed, a biopsy is taken and a frozen section is sent off for preliminary pathologic examination, which is consistent with a schwannoma. The tumor is further “undressed” by peeling away the arachnoid from the tumor capsule surface. The lateral pole of the tumor is debulked with an ultrasonic aspirator to gain access to the porus acusticus. The Tübingen line is identified to visualize the internal auditory canal. The dura over the IAC is incised with a No. 11 blade, and the dura is peeled away from the petrous bone to the lip of the porus acusticus. The posterior meatus of the IAC is then drilled away in a 270° fashion with a high-speed diamond drill with copious irrigation.

The dura over the IAC is incised sharply to expose the intracanalicular portion of the tumor. A McElveen knife is used to divide the superior and inferior vestibular nerves to identify the lateral pole of the tumor. We turn our attention to the inferior pole of the tumor and begin to dissect the tumor capsule from the arachnoid. As we peel the tumor capsule away, we can now visualize the lower cranial nerves behind the veil of arachnoid. It is important to keep this arachnoid intact and stay out of the subarachnoid space as much as possible.

We now identify the splayed inferior vestibular nerve on the surface of the tumor and begin to develop a plane between the vestibular nerve and the tumor capsule using sharp dissection and scissors spreading. This is the subperineural plane of dissection.^4^ This thin perineurium often appears as a thin veil of arachnoid-like tissue, which acts as a buffer to protect the facial and cochlear nerves on the ventral side.^5,6^ We continue the dissection along the subperineural plane distally toward the IAC. Sharp dissection is used to free up adhesions and further create this subperineural plane. The perineurium is gently dissected away using a suction sweeping technique to peel the inferior pole of the tumor away from the brainstem.

The arachnoid is now sharply incised over the superior pole of the tumor, and gentle spreading of the arachnoid away from the tumor capsule reveals the trigeminal nerve. We pick up the subperineural plane on the superior pole of the tumor, carefully separating the tumor capsule away from the superior vestibular nerve fibers. Again, sharp dissection with microscissors facilitates the development of this subperineural plane. As we dissect further along the plane, the perineurium becomes a thin arachnoid-like veil that shields the facial nerve from direct microdissection trauma.

Gentle spreading with the microforceps separates the tumor from the facial nerve at the brainstem. Intermittent debulking with the ultrasonic aspirator is performed to facilitate extracapsular dissection. The lateral end of the tumor is now dissected away from the perineurium by using a Rhoton 3 disc dissector. Here we use a “push-away” technique where the tip of the dissector rides the top of the tumor while the flat portion of the disc pushes away the perineurium. The remainder of the tumor adhesions to the perineurium is detached sharply with microscissors. Any strict tumor adhesions to the perineural layer can be trimmed away, leaving a sliver of tumor remnant to avoid facial nerve injury. We achieved a near-total resection of the tumor with residual adherent to the perineurium overlying the facial nerve. Hemostasis is achieved with a thin layer of Surgicel, and bipolar cautery should be avoided. The facial nerve stimulated at 0.05 mA at the brainstem, and the nerve took a cephalad course before turning laterally into the IAC. Waves 3 and 5 on the ABR remained intact at baseline.

It is important to wax off any petrous air cells within the IAC. In this case, we placed a piece of Bicol to protect the facial nerve, so that we could seal off the air cells with bone cement. A small fat graft is placed over the IAC defect and secured with a Surgicel cargo net, followed by some fibrin glue. This is to prevent CSF fistula into the middle ear.

### 9:51 Closure and Reconstruction

The dura is closed in a watertight fashion, and the mastoid air cells are sealed with bone wax. A fat graft is placed over the suture line, filling the craniectomy defect, and a Medpor Titan cranioplasty is used to buttress the fat graft to prevent CSF leak and pseudomeningocele. Standard multilayered wound closure is performed.

### 10:12 Postoperative Course

Postoperatively, the patient was neurologically intact with improved gait. His facial nerve function was a House-Brackmann grade I on postoperative day 1. Hearing was grossly intact initially; however, this progressed to class D hearing on a subsequent audiogram 2 months later. He is currently being considered for a cochlear implant.

### 10:32 Postoperative Imaging

Immediate postoperative MRI showed excellent decompression of the brainstem with minimal linear enhancement along the brainstem. This remained stable on follow-up MRI at 9 months without evidence of tumor recurrence. Hydrocephalus resolved without requiring a shunt.

### 10:50 Conclusions

In summary, the subperineural dissection technique is a useful strategy to preserve facial nerve function and maximize extent of tumor removal in vestibular schwannoma surgery.

## Disclosures

The authors report no conflict of interest concerning the materials or methods used in this study or the findings specified in this publication.

## Author Contributions

Primary surgeon: Liu. Assistant surgeon: Jyung. Editing and drafting the video and abstract: Liu, Tayebi Meybodi. Critically revising the work: Liu, Tayebi Meybodi. Reviewed submitted version of the work: Liu, Jyung. Approved the final version of the work on behalf of all authors: Liu. Supervision: Liu.

## References

[1] Dunn IF Bi WL Erkmen K Medial acoustic neuromas: clinical and surgical implications J Neurosurg 2014 120 5 1095 1104 24527822 10.3171/2014.1.JNS131701

[2] Silva J Cerejo A Duarte F Silveira F Vaz R Surgical removal of giant acoustic neuromas World Neurosurg 2012 77 5-6 731 735 22120302 10.1016/j.wneu.2011.08.019

[3] Yashar P Zada G Harris B Giannotta SL Extent of resection and early postoperative outcomes following removal of cystic vestibular schwannomas: surgical experience over a decade and review of the literature Neurosurg Focus 2012 33 3 E13 10.3171/2012.7.FOCUS12206 22937847

[4] Sasaki T Shono T Hashiguchi K Yoshida F Suzuki SO Histological considerations of the cleavage plane for preservation of facial and cochlear nerve functions in vestibular schwannoma surgery J Neurosurg 2009 110 4 648 655 18928359 10.3171/2008.4.17514

[5] Liu JK Dodson VN Jyung RW Retrosigmoid transmeatal approach for resection of acoustic neuroma: operative video and technical nuances of subperineural dissection for facial nerve preservation J Neurol Surg B Skull Base 2019 80 (3)(suppl 3) S269 S270 31143584 10.1055/s-0039-1688488 PMC6534509

[6] Liu JK Dodson VN Jyung RW Translabyrinthine approach for resection of large cystic acoustic neuroma: operative video and technical nuances of subperineural dissection for facial nerve preservation J Neurol Surg B Skull Base 2019 80 (3)(suppl 3) S267 S268 31143583 10.1055/s-0039-1685534 PMC6534508

